# The criteria used by private general practitioners in Bloemfontein for the diagnosis and referral of asthma patients aged 6–15 years

**DOI:** 10.4102/phcfm.v2i1.110

**Published:** 2010-06-04

**Authors:** Monia Balt, Caroline Joubert, Michelle Nel, Lincoln J. Solomon, Gina Joubert

**Affiliations:** 1 Faculty of Health Sciences, University of the Free State, South Africa; 2 Department of Paediatrics and Child Health, University of the Free State, South Africa; 3 Department of Biostatistics, University of the Free State, South Africa

**Keywords:** asthma, childhood, criteria, diagnosis, general practitioners, referral

## Abstract

**Background:**

Participants in the study were general practitioners (GPs) in private practice in Bloemfontein, South Africa.

**Objectives:**

To determine and evaluate the criteria employed by GPs in Bloemfontein to diagnose and refer chronic and acute asthma patients aged 6–15 years and to investigate the actual diagnostic criteria used by GPs, as compared to the theoretical (i.e. textbook) criteria.

**Method:**

A descriptive study was performed. A questionnaire was designed to investigate which methods of diagnosis were employed by GPs with regard to childhood asthma. The questionnaire was distributed to GPs who fulfilled certain inclusion criteria and were selected by means of simple random sampling. Statistical analysis of data was done by the Department of Biostatistics, University of the Free State, and results were summarised as frequencies and percentages.

**Results:**

Certain elements were lacking with regard to the patients’ histories taken by GPs. These included severity and frequency of attacks, as well as precipitating factors, such as smoking in the family and allergies. A worrisome number of GPs did not seem to be aware of the exact clinical picture of asthma in children and some failed to use the prescribed guidelines proposed for diagnosis of this condition in young patients. Most GPs indicated that they refer asthmatic children to private specialists, although this practice depended on the medical aid status of the patient's parents/guardian.

**Conclusion:**

As portrayed by the feedback obtained from these Bloemfontein-based GPs, it could be presumed that the diagnosis of asthma in children did not always meet the standard criteria.

## INTRODUCTION

Globally, more than 300 million people of all ages suffer from asthma, while the number of disability-adjusted life years (DALYs) lost due to asthma has been estimated to be about 15 million per year.^1^

Childhood asthma affects more than six million children in the USA.^2^ The Centers for Diseases Control and Prevention (CDC) reported that, in 2007, 8.8% and 11.4% of all children aged 5–11 years and 12–17 years, respectively, suffered from asthma.^3^ In a study conducted in Cape Town schools, Zar et al.^4^ found an increase in the prevalence of asthma in adolescents from 1995 to 2002. Asthma, next to gastroenteritis and pneumonia, is the third most common reason for hospitalisation of children in South Africa.^1^

A Cape Town study estimated the prevalence of childhood asthma at 12% – 15%. The prevalence differed for children in rural versus urban areas, with a prevalence of 0.0015% in the Transkei (rural), compared to 3.1% in children living in Guguletu, Cape Town (urban).^5^

Asthma impacts on the quality of life, poses long-term implications and may be severe. Therefore, proof of a diagnosis of asthma is required.^6^ Green and Potter^6^ proposed that criteria for a working diagnosis of asthma in children should include:

- wheezing more than once a month
- activity-induced coughing or wheezing
- coughing at night
- absence of seasonal variation
- symptoms persisting after the age of 3 years
- symptoms aggravated by certain exposures (e.g. exercise, viral infections, animal fur/hair, house dust mites, mould, smoke, pollen, changes in weather, strong emotional expression, airborne chemicals, or dust)^2^
- common colds repeatedly deteriorating to chest infections
- response to a bronchodilator and/or a 10-day course of oral steroids
- rhinitis, eczema/atopic dermatitis or food allergies in addition to symptoms of asthma
- a family history of asthma.

The five most important criteria indicated in the literature that should be taken into account for an initial diagnosis of asthma are, (1) the presence of recurrent wheezing, (2) symptomatic improvement with a bronchodilator, (3) recurrent cough, (4) exclusion of other possible diagnoses and (5) suggestive peak expiratory flow (PEF) findings.^7^ The modified bronchodilator response test (MBRT) is regarded as a diagnostic method in children, since bronchial hyperresponsiveness to a bronchodilator occurs only in asthmatics.^6^

A decrease in referrals of childhood asthma patients from the Bloemfontein community to tertiary level healthcare was observed. Records at the Universitas Academic Hospital showed an overall decline in the number of childhood asthma patients seen at this institution over a 6-year period, from 367 in 1998, to 253 in 2003. The authors suspected the most likely reason was that general practitioners (GPs) who were seeing asthmatic children at primary care level were not employing standard criteria to diagnose asthma and were therefore ‘missing’ cases which required referral. The aim of this study was to investigate the criteria used by GPs to diagnose childhood asthma.

## METHOD

A descriptive study design was used. The target population consisted of 142 private GPs in Bloemfontein who were identified by means of a commercial directory of private practitioners in South Africa. Ninety GPs were selected by simple random sampling. The initial sample size was revised because 30 of the sampled 90 general practitioners had either specialised, relocated or retired. Hence only 60 GPs qualified to be included in the study.

Inclusion criteria for participants:

- registration with the Health Professions Council of South Africa (HPCSA)
- private practitioner status
- practicing GP in Bloemfontein
- English- or Afrikaans-speaking
- agreement by GP to give informed consent to be included in the study.

Anonymous, self-administered questionnaires were delivered to the GPs’ receptionists and later collected for analysis. Because the target population was reduced, a pilot study was not considered feasible as less data would be available for analysis, thus rendering the actual study results less representative.

Approval to conduct the investigation was granted by the Ethics Committee of the Faculty of Health Sciences, University of the Free State (UFS) and the Head of Clinical Services of the Universitas Academic Hospital. The results were analysed by the Department of Biostatistics, UFS, and summarised as frequencies and percentages.

## RESULTS

Thirty-two GPs responded, giving a response rate of 53%. Their median experience (time) in private practice was 12.5 years, while their range of experience was 2–30 years.

Ninety-seven per cent of doctors indicated that they always took a thorough history of juvenile patients. The remaining 3% did not respond to this questionnaire item. Fifty per cent of GPs observed an increase in childhood asthma cases in their practice over the preceding three years, 6.3% reported a decrease, 25% said there was no change in the incidence and 18.7% were unable to determine any change. Of the 32 participants, 87.5% believed that they were adequately equipped to manage asthma in children and therefore did not refer asthmatic children to a specialist.

Questions considered by respondents to be important in obtaining a history from a child regarding asthma pertained to: cough and shortness of breath triggered by exercise (54.9%), associated atopic conditions (48.4%), family history of asthma (45.2%), night-time wheezing (42%), previous hospitalisation and frequency of attacks (22.6%), onset and duration, as well as pet and food allergies (16.1%). With regard to the family history of a child presenting with symptoms of asthma, the questions considered to be important related to: the presence of allergies, rhinitis or eczema in family members (75%), family members suffering from asthma (71.9%), smoking in the family (18.8%), information on treatment (12.5%) and the age of onset of asthma in family members (6.3%).

The methods used for the diagnosis of asthma are listed in Table 1.

**TABLE 1:** Methods used by participating GPs (*n* = 32) to diagnose asthma in children

Method	Always (%)	Sometimes (%)	Never (%)
Use of a bronchodilator	19	62	19
Mini-Wright peak flow meter	25	34	41
Simple exercise test	3	63	34
Asthma diary	28	50	22
Lung function tests	34	67	9

Of those GPs who used a bronchodilator to assist with the diagnosis of childhood asthma, 82.6% indicated that improvement in the patient's symptoms confirmed the diagnosis. Approximately 9% said that a positive response in the patient's PEF after use of a bronchodilator would help them to make the diagnosis, while 4.4% indicated that the bronchodilator should be administered by another doctor and 4.3% stated that they used it only as a screening test.

General symptoms considered by the participating GPs to be indicative of asthma in a child aged 6–15 years are summarised in Table 2.

**TABLE 2:** General symptoms considered by participants to be indicative of childhood asthma

Symptom	% of participants (*n* = 32)
Night-time and early morning cough	100
Wheezing	93.8
Tight chest	93.8
Allergic rhinitis	90.6
Cough	68.8
Post-nasal drip	40.6
Blocked nose	40.6

Nineteen per cent of participants indicated that they would always consider it beneficial to refer a child with asthma for specialist evaluation and/or care. The remaining 81% would only sometimes do so. The majority of participants (93.8%) reported that they would refer an asthmatic child to a private specialist, 37.5% would refer the child to an academic specialist clinic and 35% would refer to either a private or an academic specialist.

## DISCUSSION

The majority of respondents (87.5%) stated that they felt adequately equipped to manage asthma in children. However, whether they were truly equipped in terms of knowledge, expertise and the availability of resources, remains undecided. The results show that many of the GPs did not consistently apply guidelines such as those proposed by Green and Potter^6^ for the diagnosis of childhood asthma.

Sixteen per cent of GPs failed to obtain information on pet- and/ or food-related allergies. Such allergies are regarded as key factors in the precipitation of an asthma attack.^2, 6^ More than 75% of participants neglected to ask about the frequency of attacks, which is crucial information when the severity of the condition has to be classified and treatment and possible referral of the patient were to be considered. Despite the fact that exposure to tobacco smoking is regarded as an important precipitating factor in childhood asthma,^2, 6^ only 18.8% of participants enquired about the smoking habits of relatives when obtaining a family history. From these results it could be concluded that the GPs’ recording of the patient's history in this area was less than ideal.

With regard to the methods used by GPs to diagnose childhood asthma, only 19% of the surveyed doctors always used a bronchodilator as a diagnostic tool, which is unacceptably low as response to a bronchodilator is a simple and efficient means of assessing the patient's condition. Of those who did use a bronchodilator, 82.6% used it correctly, that is, to state that an improvement of symptoms was suggestive of asthma.

More than 40% of the respondents indicated that they never used a mini-Wright peak flow meter for the diagnosis of asthma. This was a surprising finding, as the peak flow meter is considered a fundamental diagnostic tool. However, the infrequent use of this apparatus could be attributed to a number of factors, including that the test is effort-dependent, relies on the patient's willingness and ability to exhale maximally and measures only the large airway function. The possibility that some doctors might not know how to use the apparatus correctly should also be considered.

More than a third of the respondents never used a simple exercise test to diagnose asthma in children. This represents a notable number of doctors and the fact that the test is time-consuming very likely explains why it is not often used. The measurement of the progression and severity of the disease by means of an asthma diary was used consistently by 28.1% of doctors, which is also a fairly low percentage, as the diary can be of value in monitoring asthma in the patients.

Lung function tests, considered to be the gold standard of diagnostic tests in the case of asthma, were always used by 34% of participating GPs when assessing a child with suspected asthma. It was encouraging to find that the majority of doctors used it either always (34%), or sometimes (67%) to diagnose childhood asthma.

Two incorrect symptoms were purposefully included in the questionnaire regarding the clinical picture of asthma in children: post-natal drip and blocked nose. More than 40% of GPs selected these symptoms as part of the clinical picture of asthma. This finding reflects the general perception that childhood asthma is a difficult clinical diagnosis and other conditions, such as respiratory infections, cystic fibrosis, tuberculosis and bronchopulmonary abnormalities, should be included in the differential diagnosis.^6^

A pertinent finding was that 93.8% of private GPs included in this study pointed out that, in the case of referring a patient, they would refer to private specialists. This alone may explain the decline in referral of children with asthma to a public sector asthma clinic. However, under-diagnosis of childhood asthma by GPs in Bloemfontein may still play a role, because the diagnosis must be made in order to treat and/or refer. It would thus be relevant to repeat this study in the private specialist sector to determine whether a change in the number of patients with childhood asthma being referred is also found there.

## CONCLUSION

The possibility exists that asthma in children is simply not diagnosed accurately by GPs. Many of the doctors included in the study did not consistently use the criteria recommended for the diagnosis of childhood asthma. In general, certain fundamental elements of both history-taking and examination appeared to be lacking. In addition, a notable number of doctors did not seem to be fully aware of the status of asthma in children, contributing to the ‘increased incidence, decreased referral’ paradox noted above. The most probable explanation for the decrease in referrals, however, is that most private GPs felt adequately equipped to manage childhood asthma and preferred referral to private specialists.

Some recommendations could be proposed to improve GPs’ diagnostic skills with regard to childhood asthma. Refresher courses or seminars could be presented to address important issues including, (1) clinical diagnostic criteria, (2) practical use of the mini-Wright peak flow meter and correct interpretation of its results and (3) the benefits of different diagnostic tools, such as the simple exercise test, use of a bronchodilator and keeping of an asthma diary.
